# Comparison of Physicochemical Properties of LipoParticles as mRNA Carrier Prepared by Automated Microfluidic System and Bulk Method

**DOI:** 10.3390/pharmaceutics14061297

**Published:** 2022-06-18

**Authors:** Camille Ayad, Altan Yavuz, Jean-Paul Salvi, Pierre Libeau, Jean-Yves Exposito, Valentine Ginet, Claire Monge, Bernard Verrier, Danielle Campiol Arruda

**Affiliations:** UMR 5305: Laboratoire de Biologie Tissulaire et d’Ingénierie Thérapeutique, Institut de Biologie et Chimie des Protéines, CNRS/Université Claude Bernard Lyon 1, 7 Passage du Vercors, CEDEX 07, 69367 Lyon, France; altan.yavuz@ibcp.fr (A.Y.); jean-paul.salvi@univ-lyon1.fr (J.-P.S.); pierre.libeau@ibcp.fr (P.L.); jean-yves.exposito@ibcp.fr (J.-Y.E.); valentine.ginet@ibcp.fr (V.G.); claire.monge@ibcp.fr (C.M.); bernard.verrier@ibcp.fr (B.V.)

**Keywords:** microfluidics, bulk method, hybrid nanoparticle, LipoParticles, mRNA transfection, liposomes, biodegradable polymer

## Abstract

Polymeric and/or lipid platforms are promising tools for nucleic acid delivery into cells. We previously reported a lipid–polymer nanocarrier, named LipoParticles, consisting of polylactic acid nanoparticles surrounded by cationic lipids, and allowing the addition of mRNA and cationic LAH4-1 peptide at their surface. Although this mRNA platform has shown promising results in vitro in terms of mRNA delivery and translation, the bulk method used to prepare LipoParticles relies on a multistep and time-consuming procedure. Here, we developed an automated process using a microfluidic system to prepare LipoParticles, and we compared it to the bulk method in terms of morphology, physicochemical properties, and ability to vectorize and deliver mRNA in vitro. LipoParticles prepared by microfluidic presented a smaller size and more regular spherical shape than bulk method ones. In addition, we showed that the total lipid content in LipoParticles was dependent on the method of preparation, influencing their ability to complex mRNA. LipoParticles decorated with two mRNA/LAHA-L1 ratios (1/20, 1/5) could efficiently transfect mouse DC2.4 cells except for the automated 1/5 assay. Moreover, the 1/5 mRNA/LAHA-L1 ratio drastically reduced cell toxicity observed in 1/20 ratio assays. Altogether, this study showed that homogeneous LipoParticles can be produced by microfluidics, which represents a promising platform to transport functional mRNA into cells.

## 1. Introduction

During the last few decades, messenger RNA (mRNA) nonviral vectors have been widely studied as a vaccine platform against infectious diseases [1]. However, no successful immunogenic response was reached in humans until the outbreak of the COVID-19 pandemic [2,3]. In 2020, a global consortium involving private industries, governments, and research groups was established in order to accelerate the development of vaccines against SARS-CoV-2 infection. Months later, the first two mRNA vaccines were approved by health regulatory agencies for emergency clinical use: mRNA-1273 (Moderna) and BNT162b2 (Pfizer/BioNtech, New York, NY, USA) [4,5]. It is important to emphasize that mRNA vaccines were achieved in record time thanks to the recent development of new ionizable lipids present in lipid nanoparticles (LNP) which improved mRNA delivery and biocompatibility in vivo, as well as facilitated endosomal escape [6]. Additionally, the previous knowledge of major viral proteins from coronaviruses readily revealed the mRNA sequence of the spike protein as the target antigen [7]. Since then, clinical trials using mRNA vaccines platforms have been expanded to treat other types of viral infections and cancer [8,9].

Benefits concerning the safety of mRNA vaccines over conventional vaccines explain the growing therapeutic interest of this new platform. First, mRNA translation occurs in the cytoplasm and does not require entry into the nucleus, unlike DNA-based vaccines. Thus, there is no risk of genomic integration [10,11]. Second, the manufacturing of clinical-grade mRNA molecules is based on in vitro transcription (IVT). This technology is a cell-free, rapid, and scalable process which avoids contamination with virus, bacteria, or cell-derived impurities [11,12]. Third, the mRNA molecular design and the choice of the delivery system can be used to adjust the relationship between the adequate transient in vivo half-life and specific adaptive immunity, ensuring its effectiveness with minimal side-effects. Furthermore, mRNA-based vaccines can be easily adapted to mRNA sequences encoding other antigenic proteins [12,13,14]. Naked mRNA molecules are large, hydrophilic, negatively charged, and highly sensitive to nuclease degradation. Due to these characteristics, the mRNA cannot easily pass through the cell membrane and be delivered into the cytoplasm where it can be translated into immunogenic protein. To overcome these hurdles, different types of nanoparticle delivery systems have been developed to protect mRNA from degradation, as well as enable cellular uptake and endosomal escape [15].

One of the main approaches to the formulation of mRNA is based on its complexation by electrostatic interactions with cationic components, such as lipids and polymers. Currently, LNPs are the most promising lipid-based nanoparticles used as an mRNA delivery system, as represented in the leading mRNA vaccines against SARS-CoV-2 infection [16,17]. The components of LNP formulations include an ionizable lipid, cholesterol, phospholipids, and polyethylene glycol (PEG) lipids. Despite their undeniable effectiveness, PEG lipids presented in LNP are suspected to be responsible for the manifestation of some side-effects after administration in humans and mice [18,19,20]. Thus, the development of mRNA delivery systems without a PEGylated component or mRNA loading optimization are alternatives for obtaining new, efficient, and safer vaccinal formulations. Lipid–polymer hybrid nanoparticles have been widely studied as nonviral vectors for mRNA delivery [21,22,23,24,25,26]. These hybrid nanoparticles present a polymeric core/lipid shell structure which combines the stability of the hydrophobic polymer and biocompatibility of lipid bilayers [27]. Additionally, the cationic lipid shell facilitates the adsorption of negatively charged mRNA onto the surface of nanoparticles and avoids their aggregation. These hybrid nanoparticles can be prepared by conventional bulk methods in which polymeric nanoparticles are mixed with preformed liposomes or lipid solution [28]. However, the bulk method relies on a multistep procedure and tends to have high batch-to-batch variation [29]. Different types of microfluidic devices have been developed in order to optimize the preparation method of nanoparticles [30,31]. Some important advantages of microfluidic devices over classical bulk methods are (i) the high control of experimental conditions and physical properties of the formulation, (ii) the rapid continuous flow mode of production, (iii) the batch reproducibility, and (iv) the reduction in reagent consumption and the resulting chemical waste [32]. Furthermore, microfluidic devices are available with a wide range of volumes which can be used for applications ranging from formulation development and screening (mL/min) to scale-up production under clinical Good Manufacturing Production (GMP) conditions (L/h) [33]. Today, the success of microfluidic technology is demonstrated by its application in the industrial manufacture of LNPs used as mRNA vaccines [4,15].

We recently compared the in vitro transfection efficiency of mRNAs using lipid–polymer hybrid nanoparticles, named LipoParticles (LP), and cationic liposomes, both decorated with cell-penetrating peptides (CPPs), LAH4-L1 [25]. LP were prepared through the assembly of poly(lactic acid) (PLA) nanoparticles, a biodegradable polymer approved for human use by the US Food and Drug Administration (FDA), and cationic liposomes composed of a mixture of 1,2-dioleoyl-3-trimethylammonium-propane (DOTAP) and 1,2-distearoyl-*sn*-glycero-3-phosphocholine (DSPC). This study showed that LP improved the in vitro transfection efficiency of mRNA compared with cationic liposomes. Ayad et al. suggested that the presence of the polymer core increases the rigidity of nanoparticles and that this physical modification could be the reason for the higher cell transfection efficiency observed with LP [25]. The positive impact of nanoparticle rigidity on cellular uptake and intracellular delivery is in agreement with the results presented in the literature [34,35]. Nonetheless, the method of LP production is a bulk method including the preparation of liposomes and their time-consuming purification by dialysis. In order to skip the liposome purification step and automate the method of preparation of LP, we hypothesized that a lipid shell could be formed onto PLA nanoparticles by microfluidics through the use of a lipid solution rather than preformed cationic liposomes. Thus, the aim of this work was to compare the physicochemical properties of LipoParticles prepared using the standard bulk method (LP) and an automated microfluidic system (LP_auto_). We also evaluated the effect of the LipoParticles preparation method on mRNA transfection efficiency and cytotoxicity in vitro. These experiments demonstrated that LP_auto_ produced by microfluidics presented a better homogeneity than LP, with a smaller hydrodynamic diameter and a more regular spherical shape. However, the method of preparation and the form of lipid used (preformed liposomes or lipid solution) to obtain LipoParticles impact their capacity to complex mRNA and subsequent transfection efficiency in vitro.

## 2. Materials and Methods

### 2.1. Materials

i-Particles^®^ (PLA nanoparticles) were purchased from Adjuvatis (Lyon, France). Lipids (1,2-distearoyl-*sn*-glycero-3-phosphocholine (DSPC) and 1,2-dioleoyl-3-trimethylammonium-propane (DOTAP)) were purchased from Avanti Polar (Alabaster, AL, USA). LAH4-L1 peptide (KKALLAHALHLLAL-LALHLAHALKKA) was purchased from GenScript (Piscataway, NJ, USA). Absolute anhydrous ethanol was purchased from Carlo Erba Reagents (Peypin, France), sterile pyrogen-free bidistilled water OTEC^®^ was purchased from Aguettant (Lyon, France), and nuclease-free water was purchased from Ambion (Thermo Fisher Scientific, Waltham, MA USA). Furthermore, 1× and 10× DPBS (Dulbecco’s phosphate-buffered saline, pH 7.4), culture medium (RPMI/glutamax and DMEM), fetal bovine serum (FBS), *N*-2-hydroxyethylpiperazine-*N*-2-ethane sulfonic acid (HEPES), and β-mercaptoethanol were all purchased from Gibco (Dublin, Ireland). Lipofectamine 2000^TM^ transfection reagent was purchased from Invitrogen™ via Thermo Scientific™ (Waltham, MA, USA).

### 2.2. Methods

#### 2.2.1. Preparation of LP Using the Classical Orbital Mixer Manufacturing Method

The standard method of preparing LP is to incubate preformed liposomes (formulated using a microfluidic process) together with a PLA-NP aqueous dispersion (purchased from Adjuvatis, France). The preparation of liposomes and LP was previously described [25]. Briefly, DSPC and DOTAP lipids in a molar ratio of 15:85 were dissolved in absolute ethanol at an initial concentration of 30 mM. Liposomes were then formulated with a NanoAssemblr™ benchtop instrument (NanoAssemblr™, Precision NanoSystems Inc., Vancouver, BC, Canada) equipped with a microfluidic cartridge. The aqueous phase was nuclease-free water, and all the parameters were set as defined by the manufacturer. The equipment was set up at 70 °C with an aqueous-to-ethanol flow rate ratio of 3:1 and a total flow rate of 12 mL/min. In order to remove ethanol and free lipids, the formulation was then dialyzed against 1× DPBS at pH 7.4 using a Slide-A-Lyzer^®^ Dialysis cassette with a 10 kDa cutoff (Thermo Scientific™, Waltham, MA, USA). All liposomes were stored at 4 °C until further use. The formation of LP was based on the stirring of PLA-NP and liposomes for 1 h at 70 °C using an orbital mixer (mLab scientific HCM 100-pro). After a centrifugation step (4000× *g*, 15 min and 15 °C) to remove all non-adsorbed lipids in the supernatant, the pellet was resuspended in nuclease-free water. LP were also stored at 4 °C until use.

#### 2.2.2. Preparation of LP_auto_ Using Microfluidic System (Automated Process)

Automatized LipoParticles (LP_auto_) were formulated using a NanoAssemblr™ benchtop instrument equipped with a microfluidic cartridge. The aqueous phase was composed of a dispersion of PLA-NP in nuclease-free water at the same dilution used to prepare LP. The organic phase was a lipid solution of DSPC and DOTAP in a molar ratio of 15:85 in absolute ethanol at 1.65 mM. The NanoAssemblr^TM^ benchtop equipment was set up at 40 °C with an aqueous-to-ethanol flow rate ratio of 2:1 and total flow rate of 12 mL/min. In order to remove ethanol and non-adsorbed lipids, the formulation was centrifuged at 4000× *g* and 15 °C for 15 min. The supernatant was discarded, and the pellet containing LP_auto_ was resuspended in the same volume of nuclease-free water. The LP_auto_ dispersion was stored at 4 °C until use.

#### 2.2.3. Method Validation and Lipid Quantification by HPLC–MS

##### Apparatus and Method

Analysis of DSPC and DOTAP was conducted using a Waters 2795 Alliance module including a quaternary pump, mobile phase degasser, autosampler, and column thermostat. Separation was carried out on a Luna C18 analytical column (150 × 4.6 mm i.d., 3 µm particle size, 100 Å pore size) supplied by Phenomenex. The analytical method, derived from Zhong et al. [36], consisted of a tertiary gradient, where A was 0.1% (*v*/*v*) FA in hexane, B was 0.2% (*v*/*v*) FA in methanol, and C was 0.1% (*v*/*v*) FA in water. Gradient was 88% B and 12% C for 3 min, changed to 13% A, 82% B, and 5% C over 1 min, and changed back to initial solvent mixture after 6 min. The total run time was 15 min. The column temperature was 60 °C and sample temperature was 30 °C. The flow rate was 1.4 mL/min, and a sample injection volume of 20 µL was used. The electrospray ionization mass spectrometer used to detect lipids was a Waters Micromass ZQ analyzer. Nitrogen gas was used at 450 L/h, and capillary and cone voltages were set to 3 kV and 50 V. The desolvation temperature and source temperature were 220 °C and 100 °C, respectively. Quantification was carried out using SIR mode (single ion recording) in positive mode at *m*/*z* = 790.1 for DSPC and 662.0 for DOTAP. Data were processed using Waters Empower 2 software version number 6.20.00.00 (Water Corporation, Milford, CT, USA).

##### Sample Treatment Procedure

The calibration curves of lipids were determine using diluted samples in methanol with a concentration range of 0.1 to 5 µg/mL for DSPC and 0.01 to 1 µg/mL for DOTAP. For the quantification of lipid content of LipoParticles, 200 µL of ethanol was added to 100 µL of LP or LP_auto_ suspension and mixed for 2 min using a vortex and 20 s in an ultrasonic bath. The procedure was repeated twice. The mixture was centrifuged at 15,000× *g* for 10 min at room temperature (RT). If necessary, the supernatant was diluted in methanol prior to injection into the column.

#### 2.2.4. Colloidal Physicochemical Properties

The mean hydrodynamic diameter and size distribution (i.e., polydispersity index, PdI) of naked vectors and the resulting mRNA formulations were determined by dynamic light scattering (DLS) analysis at 25 °C and at a scattering angle of 173°. All zeta potentials were measured by laser Doppler velocimetry at a scattering angle of 12.5°. The apparatus used was a Zetasizer Nano ZS (Malvern, UK). All samples were prepared by a 1/50 dilution in a 0.22 µm filtered 1 mM NaCl solution. Each value given by the software was the mean of four independent measurements.

#### 2.2.5. Transmission Electron Microscopy (TEM)

LP or LP_auto_ were used at a 0.2% solid rate and deposited on carbon/Formvar-coated copper 200 mesh grids (Spi Supplies, Structure Probe Inc., West Chester, PA, USA) for 60 s. A negative staining consisting of 1% (*w*/*v* in water) tungsten silicate solution was then applied to the grid for 30 s, and grids were washed with distilled water for 30 s. All samples were imaged using a transmission electron microscope JEOL 1400 Flash instrument at an accelerating voltage of 120 kV. All samples were imaged at 50,000× and 100,000× magnification. The particle analysis of TEM images was performed using Fiji ImageJ software. The size distribution frequency of PLA-NP and LipoParticles was evaluated using 1 µm scaled images of two independent assays and more than 200 particles.

#### 2.2.6. Adsorption of Nucleic Acids Using the Particulate Layer-by-Layer (pLbL) Strategy

The pLbL strategy, as previously described [25], relies on the successive immobilizations of anionic nucleic acids and positively charged LAH4-L1 peptide onto LipoParticles. To prepare final formulations from either LP or LP_auto_, Fluc mRNA was diluted at a concentration of 40 µg/mL in nuclease-free water and mixed (*v*/*v*) with LP or LP_auto_. Then, two volumes of LAH4-L1 peptide at a concentration of 400 µg/mL (mRNA/LAH4-L1 ratio of 1/20 *w*/*w*) or 100 µg/mL (mRNA/LAH4-L1 ratio of 1/5 *w*/*w*) were added onto the LP–mRNA intermediates. Fluc mRNA (CleanCap^®^ Fluc mRNA, (L-7602, 1.9 kB) was purchased from TriLink BioTechnologies (San Diego, CA, USA).

#### 2.2.7. mRNA Complexation Assay

The complexation of mRNA onto LP and LP_auto_ was evaluated using an electrophoretic mobility shift assay. To this aim, a 1% agarose gel was prepared in Tris–borate–EDTA (TBE) 1× buffer, and ethidium bromide (EtBr) was added as staining. Formulations were either treated or not with heparin (Sanofi-Aventis, Ploermel, France) at RT for 30 min plus with proteinase K (NEB, Évry-Courcouronnes, France) at 56 °C for 15 min. The gel was then loaded with samples mixed with 2× loading dye (2× solution of 95% formamide, 18 mM EDTA, 0.025% SDS, xylene cyanol, and bromophenol blue; Invitrogen, Carlsbad, CA, USA) containing 100 ng of mRNA per well. The electrophoresis was performed for 17 min at 100 V, and the stained mRNA bands were visualized on an ultraviolet transilluminator and digitalized.

#### 2.2.8. Cell Culture

Immortalized DC2.4 (murine bone marrow-derived dendritic cells) cells were obtained from InvivoGen (Toulouse, France). They were cultured in RPMI-1640 medium, supplemented with 10% heat-inactivated FBS, 10 mM Hepes, and 50 µM β-mercaptoethanol. Cells were cultured in a 37 °C incubator (Heracell 150i, Thermo Scientific) under 5% CO_2_ and 95% humidity. Cells were always used with a low passage number.

#### 2.2.9. mRNA Transfection Efficiency on Culture Cells

One day prior to transfection, cells were seeded in a 96-well plate at a density of 20,000 cells per well. After 24 h, complete medium was removed and replaced by a 100 µL mix of formulation and medium without serum, allowing the transfection of 90 ng of Fluc mRNA or equivalent volumes of noncomplexed LP and LP_auto_. Supernatants were removed 3 h post transfection, and 100 μL of complete medium was added to each well. Finally, cells were incubated at 37 °C and 5% CO_2_ until the analysis 24 h later. Lipofectamine 2000^TM^ transfection reagent was used as a positive control following the manufacturer’s instructions. Non-transfected cells were used as a negative control and were labeled as ‘untreated’.

A luciferase assay was performed using the Bright-Glo™ Luciferase Assay System (Promega, Charbonnières-les-Bains, France) according to the manufacturer’s recommendations. Briefly, the luciferine substrate was added (*v*/*v*) to the well and left to incubate for 5 min at RT. Luminescence was then measured on a Tecan i-control Infinite M1000 (Integration time 1 s) (Tecan, Männedorf, Switzerland) and was determined as the mean of three replicates and three independent experiments.

#### 2.2.10. Cytotoxicity toward Culture Cells

The cytotoxicity of noncomplexed vectors and Fluc mRNA LP and LP_auto_ formulations was evaluated 24 h post transfection (as described above) using a PrestoBlue™ Assay (Thermo Scientific™, Waltham, MA, USA) according to the manufacturer’s instructions. Briefly, 11 µL of PrestoBlue™ Cell Viability Reagent was added to the wells, and plates were incubated for 20 min at 37 °C. Fluorescence was detected on a Tecan i-control Infinite M1000 (560 nm/590 nm; bandwidth 10 nm) instrument (Tecan, Männedorf, Switzerland). Fluorescence was determined as the mean of three replicates and three independent experiments.

#### 2.2.11. Statistical Analysis

Statistical analyses were performed using GraphPad Prism version 9.3 software (San Diego, CA, USA). All the data are presented as the mean ± SD. Differences between groups were analyzed using one-way ANOVA for the cytotoxicity and two-way ANOVA for the transfection efficiency, both followed by Tukey’s multiple comparison test. Statistical significances are indicated on the figures.

## 3. Results

### 3.1. Characterization of LipoParticles Obtained from Two Different Manufacturing Methods: Manual and Automated

**Figure 1:**
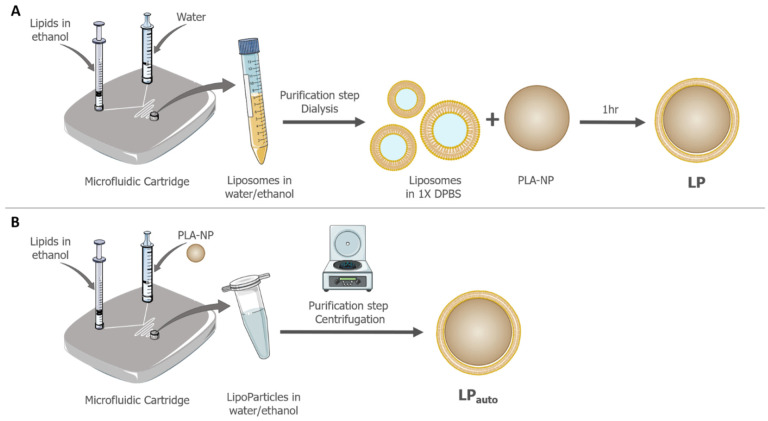
Preparation of LipoParticles (**A**) using the standard bulk method or (**B**) through an automated mixing using microfluidic system.

LP, which consist of PLA-NP coated with lipid bilayers, were already previously described and studied for the delivery of mRNA [25]. Here, the aim was to evaluate the transposition of the manufacturing process of LP from a bulk method to an automated microfluidic system. The two methods used are depicted in Figure 1. The manual method was based on the incubation of preformed liposomes, obtained by microfluidics and requiring a dialysis purification step, and a PLA-NP dispersion in the orbital mixer (Figure 1A). For the full automated production, the PLA-NP dispersion and lipid solution were mixed into the microfluidic cartridge and purified in one step through centrifugation (Figure 1B). Even if our objective was not to develop a new formulation, we evaluated the effect of the lipid solution concentration injected as an organic phase in the microfluidic system on LP_auto_ physicochemical characteristics. Briefly, LP_auto_ were prepared with a lipid solution at the same DSPC/DOTAP ratio and following concentrations: 0.3, 0.9, 1.5, 1.65 (equivalent to the lipid concentration of liposomes when preparing LP using the bulk method) and 3 mM. When the lipid concentration was lower than 1.65 mM, the ζ potential was inferior to +20 mV and considered as an unstable colloidal dispersion. When LP_auto_ were synthetized with lipid solutions at 1.65 and 3 mM, no differences in hydrodynamic diameter and ζ potential were observed between the two conditions (data not shown). Additionally, no difference in DLS and zeta potential of LP_auto_ was observed when prepared at two total flow rate (TFR) conditions of the microfluidic system, 10 and 12 mL/min (data not shown). Considering these preliminary evaluations of LP_auto_ production, the condition chosen for the microfluidic system was a TFR of 12 mL/min and the injection of an organic phase composed of a DSPC/DOTAP 15:85 (*m*/*m*) solution at 1.65 mM, which again corresponded to the lipid concentration of liposomes used to prepare LP by bulk method and, thus, enabled a fairer comparison of both LP. In both methods, LipoParticles were prepared from the same PLA-NP batch, presenting a hydrodynamic diameter of approximately 145 nm and a negative surface charge close to −46 mV (Table 1).

To compare the physicochemical properties of LipoParticles prepared using both methods, their size (hydrodynamic diameter) and ζ potential were first assessed using DLS and zetametry, respectively (Table 1). The preparation using the microfluidic system led to a decrease in the mean hydrodynamic diameter from 226 to 175 nm for LP and LP_auto_, respectively, with similar PdI, independently of the method of preparation. As expected, an inversion of the surface charge was observed from anionic PLA-NP to cationic LP and LP_auto_. This clearly evidenced the surface modification of PLA-NP by positively charged liposomes in the case of LP or cationic lipids directly in the case of LP_auto_.

**Figure 2:**
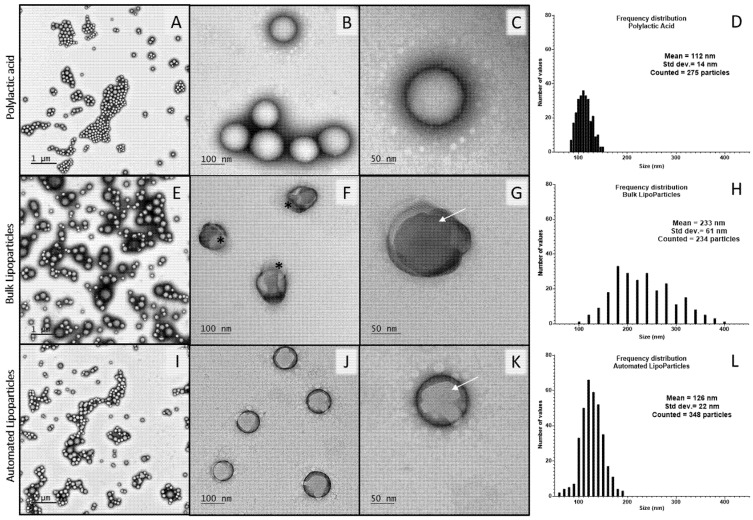
TEM images of PLA-NP (**A**–**C**), LP (**E**–**G**), and LP_auto_ (**I**–**K**) stained with 1% (*w*/*v* in water) tungsten silicate solution. White arrows indicate lipid layers on LipoParticles (**G**,**K**), while black asterisks indicate eccentric lipid vesicles attached to LP (**F**). Size particle analysis was performed on 1 µm scaled images to evaluate the size distribution frequency of nanoparticles (graphs **D**,**H**,**L**). Scale bars are indicated on each micrograph.

The morphology of mRNA vectors was observed by TEM with a negative staining to identify the lipid deposition. Both bulk and automated LipoParticles (Figure 2G,K, respectively) preserved the spherical shape of PLA-NP used as a solid core (Figure 2C). TEM analysis also highlighted the presence of multilamellar streaks onto LP and LP_auto_ (white arrows in Figure 2G,K, respectively) suggesting the presence of lipid layers since they were not observed in PLA-NP. Some multilamellar structures showed an eccentric association to LP prepared using the bulk method (black asterisks in Figure 2F), whereas, when using a microfluidic system, LP_auto_ presented increased homogeneity with the concentric lipid deposition (Figure 2J). This could explain the difference in negative staining contrast, more intense on LP than LP_auto_. The analysis of size distribution frequency using TEM images showed that the mean diameters of LP (Figure 2H) and LP_auto_ (Figure 2L) were 233 and 126 nm, respectively. In accordance with DLS data, both LipoParticles presented a higher size than PLA-NP used as the polymeric core (112 nm, Figure 2D).

In order to quantify the amount of lipid complexed to each type of LP, an HPLC–MS method was developed for both DOTAP and DSPC and validated according to ICH guidelines [37]. As shown in Table 2, the percentage error (PE) from the theoretical concentration and relative standard deviation (RSD) were calculated to present the accuracy and precision of the method. The regression line equations calculated by the least-squares method were *y* = 35,166,786*x* + 614,485 with a correlation coefficient (*r*) of 0.999 for DSPC and *y* = 188,945,499*x* + 450,927 with *r* = 0.997 for DOTAP.

The results are presented in Table 3. For both LP and LP_auto_, the initially chosen molar ratio of 15/85 DSPC and DOTAP was maintained throughout the stages of each manufacturing process, with final proportions of 13/87 and 17/83 for LP and LP_auto_, respectively. However, the total concentration of lipids was found to be significantly lower (more than fourfold lower) for LP_auto_ obtained in a single step than for LP.

### 3.2. Complexation of mRNA and LAH4-L1 on LP and LP_auto_ Using pLbL Approach

Since LP and LP_auto_ vectors are mainly considered for mRNA delivery, their ability to adsorb such molecules was then investigated. For this purpose, we used the previously implemented and described pLbL formulation strategy [25]. Briefly, this strategy consisted of using electrostatic interactions to successively immobilize nucleic acids on positively charged LipoParticles and then LAH4-L1, a cell-penetrating peptide, on negatively charged LipoParticles-mRNA intermediates. For this study, an mRNA-encoding luciferase enzyme was used, and two different ratios of mRNA/LAH4-L1 were evaluated: 1/5 and 1/20 *w*/*w*.

The hydrodynamic diameter of formulations prepared with LP or LP_auto_ were analyzed by DLS (Table 4). The addition of mRNA and LAH4-L1 induced an increase in size, compared with the corresponding initial LipoParticles (226 to 280 nm for LP, 175 to 225 nm for LP_auto_). The increase in PdI resulted from a slight heterogeneity of the formulations and may reflect the presence of particles with different sizes or some aggregates. To note, LP_auto_ led to a more homogeneous final formulation than LP prepared using the classical method when a 1/5 *w*/*w* mRNA/LAH4-L1 ratio was used, whereas no such difference was observed between formulations from LP or LP_auto_ at a 1/20 *w*/*w* ratio. Lastly, the zeta potential dropped in all formulations compared to unloaded LP and LP_auto_, suggesting an efficient immobilization of mRNA and then peptides on LP and LP_auto_. In addition, the surface charge varied with the amount of peptide added, while the surface charge increased by 13–14 mV with a 1/20 *w*/*w* ratio of mRNA/LAH4-L1 compared with the 1/5 ratio.

**Figure 3:**
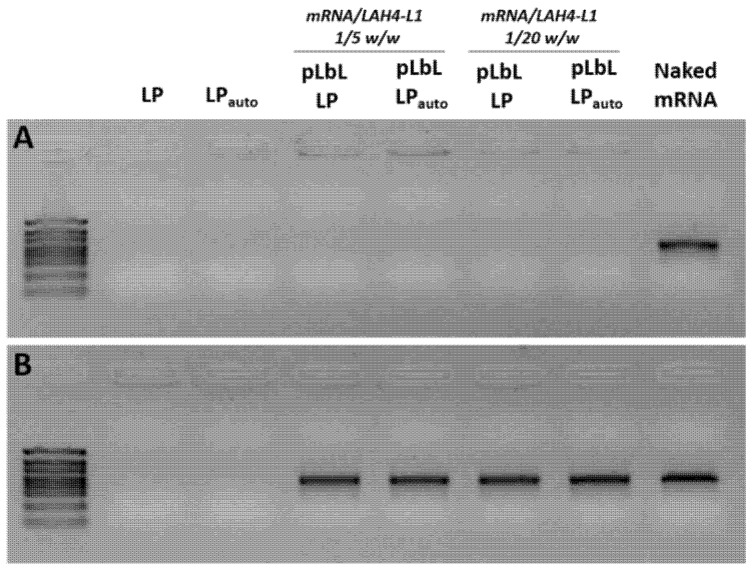
Agarose gel electrophoresis assays of pLbL formulations (**A**) without and (**B**) with mRNA desorption treatment. The concentration of Fluc mRNA was 10 µg/mL.

To further verify the ability of LP_auto_ to bind mRNA using the pLbL strategy, an agarose gel electrophoresis assay was performed. Regardless of the LP manufacturing process used and the mRNA/LAH4-L1 ratio, mRNA was fully complexed, as no free mRNA was observed on the gel (Figure 3A). Moreover, a desorption treatment (heparin/proteinase K) was carried out on formulations to verify the integrity of mRNA complexed to LP and LP_auto_. As shown in Figure 3B, the electrophoretic mobility of mRNA was identical to that of free mRNA regardless of the carrier used. These results clearly demonstrate that the pLbL strategy, which was already applied for the complexation of mRNA with classical LP, can be used with LP_auto_, since it allowed efficient complexation of the mRNA without causing its degradation.

**Figure 4:**
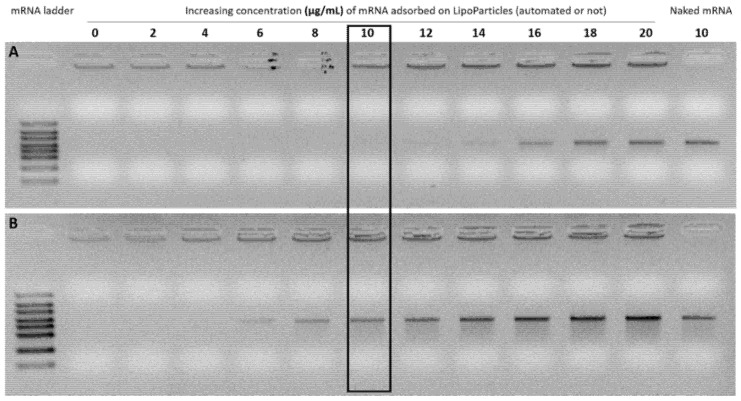
Determination of the amount of mRNA that can be adsorbed onto (**A**) LP and (**B**) LP_auto_ using agarose gel electrophoresis assays. To this aim, formulations containing increasing concentrations of mRNA were prepared and directly deposited on 1% agarose gel. The black box represents the mRNA concentration used in the pLbL formulations.

Considering that LP_auto_ had a lower total lipid concentration than LP and that mRNA molecule complexation is directly correlated to the positive charges of cationic lipids, we then investigated whether lipid content could have an impact on the mRNA adsorption onto both LipoParticles. In this regard, increasing concentrations of mRNA were adsorbed onto each carrier, and an electrophoretic mobility shift assay was performed. As shown in Figure 4, the maximum mRNA immobilization capacity on the LP surface was reached when the free mRNA band corresponding to naked mRNA appeared on the gel. While it was possible to completely adsorb mRNA up to a concentration of 12 µg/mL with LP, the adsorbed amount was at least twofold lower with LP_auto_ since a free mRNA band was visible from 6 µg/mL of mRNA.

### 3.3. In Vitro Investigation of pLbL Formulations with Transfection and Cytotoxicity Studies

**Figure 5:**
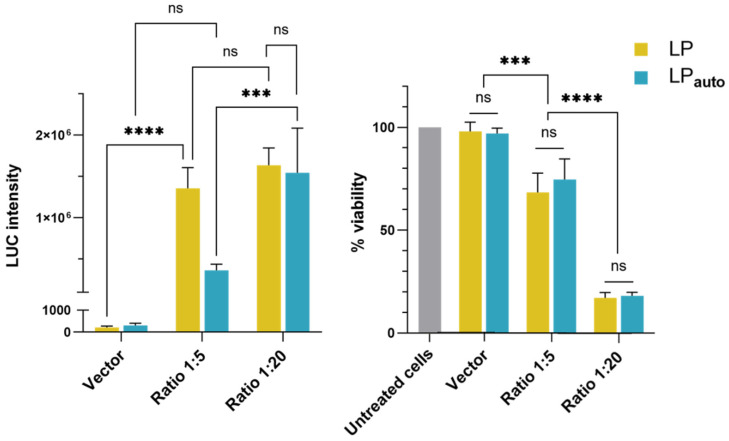
In vitro evaluation of (**left**) transfection efficiency using Bright-Glo luciferase assay and (**right**) cell viability (PrestoBlue assay) of pLbL formulations. Transfections were performed on DC2.4 cells. Measurements were always performed 24 h post transfection. Data are presented as the mean ± SD (not significant (ns): *p* > 0.05, ***: *p* < 0.001, ****: *p* < 0.0001).

In the next step, we investigated the in vitro transfection efficiency of LP or LP_auto_ complexed with 10 µg/µL of mRNA on DC 2.4 cells. As shown in Figure 5 (graph on the left), the transfection profiles were different between the two types of LP depending on the mRNA/LAH4-L1 ratio. A very efficient expression of mRNA was observed when pLbL formulations were prepared at a 1/20 *w*/*w* mRNA/LAH4-L1 ratio, even if it was significantly lower than the FLuc expression obtained with the commercial transfecting agent Lipofectamine 2000^TM^ used as a control (data not shown). Furthermore, there was no significant difference between transfection realized with LP obtained via the bulk method or the microfluidic system at this ratio. However, when the amount of peptide in the formulation was decreased until an mRNA/LAH4-L1 ratio of 1/5, LP_auto_ was significantly less efficient for mRNA transfection than conventional LP. While there was no significant difference observed between the 1/5 and 1/20 mRNA/LAH4-L1 ratio for LP, LP_auto_ showed a reduced transfection efficiency when peptide loading in the pLbL formulation was decreased. Regarding cytotoxicity (Figure 5, graph on the right), both naked vectors were found to be safe on DC cells in contrast to the resulting pLbL formulations. The systematic cytotoxicity observed with pLbL formulations in DC2.4 cells was previously studied and was to be inherent to the LAH4-L1 peptide [25]. Thus, by reducing the peptide intake in the formulation from a 1/20 *w*/*w* mRNA/LAH4-L1 ratio to 1/5 *w*/*w*, cell viability was significantly improved from 17% to 69% and from 18% to 75% for LP and LP_auto_, respectively. It should be mentioned, however, that the pLbL formulations remained more toxic than the vectors alone.

## 4. Discussion

The application of microfluidic systems has been widely used to manufacture different types of nanocarriers for the delivery of drugs and nucleic acids such as the LNP formulation used in mRNA vaccines against SARS-CoV-2 [1], liposomes [38,39], and polymeric [40,41,42,43,44] and hybrid nanoparticles [45,46]. In this work, we were able to produce a lipid–PLA hybrid nanocarrier, named LipoParticles, with improved homogeneity using an automated microfluidic system (LP_auto_) compared to LipoParticles prepared previously using the conventional bulk method (LP) [25]. In the case of LipoParticles formed using the conventional bulk method, the ratio between the surface areas of lipid vesicles and polymeric particles, Av/Ap, was the major parameter affecting their assembly [47]. Since the synthesis of LP_auto_ was performed from lipids solubilized in ethanol, Av/Ap could not be applied, as lipid vesicles or liposomes were not preformed separately. Zhang et al. [48] demonstrated that the physicochemical properties of hybrid nanoparticles could be modified by varying the polymer/lipid ratio (*w*/*w*) using a microfluidic chip. In order to maintain a correlation between the composition of the lipid solution and liposomes used for the preparation of LP_auto_ and LP, respectively, we established that the microfluidic system would be loaded with a solution of DSPC/DOTAP at an equivalent molar ratio and concentration of liposomes used to prepare LP via the standard bulk method. However, the temperature used in the preparation with the microfluidic system could not be maintained at 70 °C as used for the liposome synthesis because the injection of PLA-NP lead to some precipitation and partial loss of components inside the channel of microfluidic cartridge. Then, the temperature was decreased to 40 °C to prevent the partial clogging of the system and ensure the total recovery of components and the formation of reproducible batches of LP_auto_. As illustrated in Figure 1, we skipped the preparation and purification of liposomes used in the bulk method via the simple complexation of a DSPC/DOTAP solution with PLA-NP to obtain LP_auto_ using the microfluidic system. It is important to indicate that, prior to physicochemical analyses, both LP were purified by centrifugation after synthesis in order to eliminate non-absorbed free lipids, lipid vesicles, and/or liposomes. Despite the same lipid–polymer composition, LipoParticles formulated using both methods presented quite different physicochemical properties and morphology.

The DLS evaluation showed that the size of LP_auto_ decreased when compared to LP, indicating that the method of preparation influences the auto assembly of lipids (Table 1). Additionally, TEM images (Figure 2) revealed that LP presented apparent higher size dispersity than LP_auto_. Compared to DLS analysis, TEM images showed the same tendency of mean size: PLA-NP < LP_auto_ < LP (Figure 2D,H,L). However, for all samples, the mean size of nanoparticles observed by TEM was lower than that observed by DLS. The divergence of data between the two techniques is probably due to the sample preparation and treatment as already identified for other types of nanoparticles [49,50]. Furthermore, the streaks observed in LP and LP_auto_ are characteristic of the contrast difference representing the deposition of multilamellar lipid structures at the PLA-NP surface. Nevertheless, LP (Figure 2B) exhibited a more irregular shape compared to the defined spherical form of LP_auto_ (Figure 2C). In accordance with our presumption that the microfluidic system allows constant and uniform mixing of components, lipid layers in LP_auto_ were apparently more homogeneously distributed around nanoparticle surface. Thevenot et al. [47] presented similar results with LP prepared using the standard bulk method from a mixture of PLA-NP and zwitterionic lipid vesicles. They proposed that lipid/particles assemblies are organized as a function of the strength of interactions between the two components. Depending on the composition of vesicles, i.e., purely zwitterionic or with the inclusion of cationic lipids, PLA-NP were covered homogeneously with lipid multilayers originated from vesicle disruption or surrounded with intact lipid vesicles. According to our results, the strength of electrostatic interaction between excess cationic liposomes and anionic PLA-NP seemed to partially disrupt liposomes and induce their reorganization in a mixture of lipid bilayers and concentric multilamellar vesicles.

Considering these differences, we assumed that the amount of lipid associated to LipoParticles was different when prepared using the bulk and microfluidic methods, even if the total concentration of lipids in solution or liposomes was the same for LP_auto_ and LP preparation, respectively. Then, the lipid content of LP and LP_auto_ was evaluated by HPLC–MS. Interestingly, the lipid ratio was very similar to the initial DSPC/DOTAP 15:85 molar ratio independently of the method of preparation using a lipid solution or preformed liposomes (Table 3). However, the total lipid concentration was more than four times higher in LP compared to LP_auto_. These results are in accordance with the higher size and positive zeta potential of LP compared to LP_auto_. Zhang et al. [48] showed that, using a two-stage microfluidic chip, they were able to control the number of lipid layers in poly(lactide-*co*-glycolide) (PLGA)–lipid hybrid nanoparticles according to the order of addition of components. Using this system, they could prepare hybrid nanoparticles exhibiting only a lipid monolayer (ML-NP) or lipid bilayers (BL-NP), in which the latter required twice the amount of lipids compared to ML-NP to completely cover the PLGA core. In the case of BL-NP, liposomes were formed in the first stage prior to the injection of PLGA solution in a second stage. For ML-NP, PLGA-NP were formed in the first stage, and then lipid solution was added. Meanwhile, they used only zwitterionic lipids that exhibited a different type of interaction with PLGA compared to the electrostatic interactions between preformed PLA-NP and cationic lipids or liposomes used in our study. These results corroborate our findings, indicating that the quantity of lipid deposition in hybrid polymer–lipid nanoparticles depends on the method of preparation and the form of lipids added, in solution or preformed liposomes, to interact with PLA-NP.

The ability of LipoParticles to complex with mRNA molecules is directly related to the electrostatic interaction between anionic phosphates from mRNA and positively charged amines from cationic lipids at the LipoParticles surface. The analysis of lipid components using HPLC–MS showed that LP_auto_ had a lower total lipid concentration than LP and, thus, a reduction in cationic lipids available for mRNA adsorption. Then, we evaluated whether LP_auto_ had equivalent properties as an mRNA carrier, as already demonstrated for LP [25]. Using the pLbL approach based on electrostatic interactions, we complexed LP and LP_auto_ with the same quantities of mRNA and cell-penetrating peptide, LAH4-L1, in a sequential manner as a function of the electrostatic interactions of charged components. LAH4-L1 is a CPP which is widely used for the transport and release of mRNAs into the cytoplasm. CPPs allow spontaneous interactions with anionic mRNA and lipid cell membranes; moreover, they have pH-sensitive residues that facilitate endosomal escape [51]. However, the complexation of LP with mRNA and LAH4-L1 led to a higher accumulation of LipoParticles in DC 2.4 cells and reduced their viability [25]. Considering our previous results, pLbL LP and pLbL LP_auto_ at 1/5 and 1/20 mRNA/LAH4-L1 ratios were chosen in order to assess whether the different vectors could modify the balance between cytotoxicity and transfection efficiency of formulations. Both formulations were successfully achieved presenting a high positive zeta potential and similar dispersion homogeneity. Not surprisingly, the hydrodynamic diameter of pLbL LP was slightly higher than that of pLbL LP_auto_ (Table 4). As with pLbL LP, mRNA was completely complexed in pLbL LP_auto_, and their integrity was preserved after undergoing desorption treatment (Figure 3). However, we observed that LP were capable of adsorbing more than twice the amount of mRNA compared to LP_auto_ before the addition of LAH4-L1, probably due to the significantly lower concentration of total lipids in the latter (Figure 4). LP_auto_ were able to complex only a part of mRNA at concentration of 10 µg/mL (Figure 4), and the remaining free molecules were likely to be complexed once the LAH4-L1 peptide is added (Figure 3). Bose et al. [52] demonstrated that, by increasing the concentration of cationic lipid from 6% to 24% in lipid–polymer hybrid nanospheres, the ability to incorporate plasmid DNA (pDNA) was significantly improved. Additionally, they showed that the inclusion of protamine as an additional cationic component into hybrid nanoparticles resulted in higher pDNA complexation in formulations. Here, we observed a similar behavior in which LP with a higher lipid concentration than LP_auto_ ensured the total complexation of mRNA. Furthermore, the addition of the cationic peptide LAH4-L1 was indispensable to obtain the complete mRNA complexation with LP_auto_ via the pLbL strategy. Since LAH4-L1 was always in excess compared to mRNA, we can hypothesize that the complexation of mRNA and LAH4-L1 at the surface of pLbL LP_auto_ formulations could be different compared to pLbL LP formulations, and mRNA transfection efficacy might be affected.

Lastly, we evaluated the mRNA expression and cytotoxicity in vitro of pLbL LP and pLbL LP_auto_ formulations at 1/5 and 1/20 mRNA/LAH4-L1 ratios. Regarding the cytotoxicity, both LP and LP_auto_ demonstrated high cell viability and safety profiles to be used as mRNA carrier. Both formulations, pLbL LP and pLbL LP_auto_, at 1/20 mRNA/LAH4-L1 ratio were able to induce efficient mRNA expression in vitro, and no significant difference was observed between both formulations (Figure 5). However, in this condition, both pLbL formulations presented high cytotoxicity (~20% cell viability) toward DC 2.4 cells, which is inherent to the presence of LAH4-L1. Our previous results showed that, by reducing the mRNA/LAH4-L1 ratio to 1/5 for pLbL LP formulations, a better balance between mRNA expression and cytotoxicity was obtained [25]. Therefore, the transfection of DC2.4 cells with pLbL LP and pLbL LP_auto_ formulations at a 1/5 mRNA/LAH4-L1 ratio confirmed that cytotoxicity was decreased, with 70–80% cell viability for both formulations. Nevertheless, we observed that mRNA expression was strongly reduced for pLbL LP_auto_ formulations, while no significant difference was observed for pLbL LP formulations at the two mRNA/LAH4-L1 ratios evaluated. Considering the same amount of mRNA used in transfection essays, we hypothesized that the reduced lipid content assembly at the surface of PLA-NP in the case of LP_auto_ could be responsible for (i) the reduced cell internalization of nanoparticles and/or (ii) the modified mRNA release and translation in vitro. To verify if there is a relationship between the physicochemical properties and in vitro activity of LipoParticles, an in-depth characterization of component organization, nanoparticle uptake pathways, and kinetics of mRNA expression needs to be considered in future studies.

## 5. Conclusions

The present study showed that LipoParticles can be prepared in a reproducible manner through an automated microfluidic system (LP_auto_). These LipoParticles presented adequate physicochemical characteristics to be used as an mRNA carrier. However, the lipid shell formed was not equivalent to that obtained via the bulk method using preformed liposomes (LP). This difference could drastically reduce the amount of mRNA adsorbed onto the PLA-NP surface. Consequently, the pLbL LP_auto_ formulation, obtained from the adsorption of mRNA and LAH4-L1 at a 1/5 (*w*/*w*) ratio using the pLbL strategy onto LP_auto_, exhibited reduced mRNA expression in vitro compared to pLbL LP prepared using the bulk method. In summary, our results showed that using a microfluidic system is a promising strategy for the synthesis of LipoParticles; however, additional evaluation of formulation parameters and microfluidic conditions needs to be performed to ensure the formation of a lipid shell able to adsorb a higher quantity of mRNA and improve its expression with low cytotoxicity.

## Figures and Tables

**Table 1:** Physicochemical characterization of PLA-NP, DSPC/DOTAP liposomes, LP, and LP_auto_ through DLS and zetametry measurements. Results are presented as the mean ± SD of different samples (*n* = 3).

Samples	Diameter (nm)	Polydispersity (PdI)	Ζeta Potential (mV)
PLA-NP	145 ± 2	0.056 ± 0.028	−46 ± 1
Liposomes DSPC/DOTAP	80 ± 7	0.126 ± 0.016	+50 ± 2
LP	226 ± 3	0.124 ± 0.004	+52 ± 3
LP_auto_	175 ± 3	0.103 ± 0.04	+43 ± 3

**Table 2:** Validation methods for DSPC and DOTAP quantification by HPLC–MS. The table presents intra-day and inter-day precision and accuracy.

	PE (%)	RSD (%)	PE (%)	RSD (%)	PE (%)	RSD (%)	PE (%)	RSD (%)
**DSPC**	**LOQ (0.1 µg/mL)**		**0.5 µg/mL**		**2 µg/mL**		**5 µg/mL**	
Intra-day ^1^	6	3.6	7	1.9	5.9	4.3	6.2	3.1
Inter-day ^2^	10.6	14.1	7	7.7	2.7	4.9	1.1	1.1
**DOTAP**	**LOQ (0.01 µg/mL)**		**0.05 µg/mL**		**0.2 µg/mL**		**1 µg/mL**	
Intra-day ^1^	12.7	9.3	2	5.2	3.9	5	3.6	1.4
Inter-day ^2^	1.6	10.4	7.8	11.4	11.5	19.6	0.3	1.1

LOQ: limit of quantification; PE: percentage error; RSD: relative standard deviation. **^1^** *n* = 8, **^2^** *n* = 5.

**Table 3:** Quantification of DSPC and DOTAP lipids contained in LP or LP_auto_. Values given correspond to the quantification of both lipids in the pellet (after removal of supernatants, i.e., removal of free lipids). Results are presented as the mean ± SD of samples (*n* = 3).

Samples	DSPC (mM)	DOTAP (mM)	Total Lipid Concentration (nM)	DSPC/DOTAP (Molar Ratio)
LP	0.012 ± 0.001	0.078 ± 0.008	0.090	13/87
LP_auto_	0.0037 ± 0.0006	0.018 ± 0.0010	0.022	17/83

**Table 4:** Physicochemical characterization of pLbL formulations with either LP or LP_auto_ as carriers through DLS and zetametry measurements. The formulations were prepared with Fluc mRNA, and the ratio of mRNA/LAH4-L1 was set at 1/20 or 1/5 *w*/*w*. Results are presented as the mean ± SD of samples (*n* = 3).

Samples	Diameter (nm)	Polydispersity (PdI)	Ζeta Potential (mV)
pLbL LP 1/5	274 ± 11	0.184 ± 0.018	+19 ± 1
pLbL LP 1/20	281 ± 24	0.202 ± 0.029	+33 ± 3
pLbL LP_auto_ 1/5	222 ± 2	0.149 ± 0.014	+16 ± 1
pLbL LP_auto_ 1/20	227 ± 20	0.197 ± 0.046	+29 ± 2

## Data Availability

Not applicable.

## References

[1] Dolgin E. (2021). The tangled history of mRNA vaccines. Nature.

[2] Kulkarni J., Cullis P.R., van der Meel R. (2018). Lipid Nanoparticles Enabling Gene Therapies: From Concepts to Clinical Utility. Nucleic Acid Ther..

[3] Maruggi G., Zhang C., Li J., Ulmer J.B., Yu D. (2019). mRNA as a Transformative Technology for Vaccine Development to Control Infectious Diseases. Mol. Ther..

[4] (2020). Messengers of hope. Nat. Biotechnol..

[5] Dolgin E. (2021). How COVID unlocked the power of RNA vaccines. Nature.

[6] Hou X., Zaks T., Langer R., Dong Y. (2021). Lipid nanoparticles for mRNA delivery. Nat. Rev. Mater..

[7] Verbeke R., Lentacker I., De Smedt S.C., Dewitte H. (2021). The dawn of mRNA vaccines: The COVID-19 case. J. Control. Release.

[8] Wang Y., Zhang Z., Luo J., Han X., Wei Y., Wei X. (2021). mRNA vaccine: A potential therapeutic strategy. Mol. Cancer.

[9] (2019). RNA Technologies Expand Tool Kit for Cancer Immunotherapy. Cancer Discov..

[10] Wadhwa A., Aljabbari A., Lokras A., Foged C., Thakur A. (2020). Opportunities and Challenges in the Delivery of mRNA-Based Vaccines. Pharmaceutics.

[11] Rosa S.S., Prazeres D.M.F., Azevedo A.M., Marques M.P.C. (2021). mRNA vaccines manufacturing: Challenges and bottlenecks. Vaccine.

[12] Sahin U., Karikó K., Türeci Ö. (2014). mRNA-based therapeutics—Developing a new class of drugs. Nat. Rev. Drug Discov..

[13] Linares-Fernández S., Lacroix C., Exposito J.-Y., Verrier B. (2019). Tailoring mRNA Vaccine to Balance Innate/Adaptive Immune Response. Trends Mol. Med..

[14] Jackson N.A.C., Kester K.E., Casimiro D., Gurunathan S., DeRosa F. (2020). The promise of mRNA vaccines: A biotech and industrial perspective. NPJ Vaccines.

[15] Kim J., Eygeris Y., Gupta M., Sahay G. (2021). Self-assembled mRNA vaccines. Adv. Drug Deliv. Rev..

[16] Greaney A.J., Loes A.N., Gentles L.E., Crawford K.H., Starr T.N., Malone K.D., Chu H.Y., Bloom J.D. (2021). Antibodies elicited by mRNA-1273 vaccination bind more broadly to the receptor binding domain than do those from SARS-CoV-2 infection. Sci. Transl. Med..

[17] Turner J.S., O’Halloran J.A., Kalaidina E., Kim W., Schmitz A.J., Zhou J.Q., Lei T., Thapa M., Chen R.E., Case J.B. (2021). SARS-CoV-2 mRNA vaccines induce persistent human germinal centre responses. Nature.

[18] Wadman M. (2020). Public needs to prep for vaccine side effects. Science.

[19] Ndeupen S., Qin Z., Jacobsen S., Bouteau A., Estanbouli H., Igyártó B.Z. (2021). The mRNA-LNP platform’s lipid nanoparticle component used in preclinical vaccine studies is highly inflammatory. iScience.

[20] Bigini P., Gobbi M., Bonati M., Clavenna A., Zucchetti M., Garattini S., Pasut G. (2021). The role and impact of polyethylene glycol on anaphylactic reactions to COVID-19 nano-vaccines. Nat. Nanotechnol..

[21] Su X., Fricke J., Kavanagh D.G., Irvine D.J. (2011). In Vitro and in Vivo mRNA Delivery Using Lipid-Enveloped pH-Responsive Polymer Nanoparticles. Mol. Pharm..

[22] Yasar H., Biehl A., De Rossi C., Koch M., Murgia X., Loretz B., Lehr C.-M. (2018). Kinetics of mRNA delivery and protein translation in dendritic cells using lipid-coated PLGA nanoparticles. J. Nanobiotechnol..

[23] Kong N., Tao W., Ling X., Wang J., Xiao Y., Shi S., Ji X., Shajii A., Gan S.T., Kim N.Y. (2019). Synthetic mRNA nanoparticle-mediated restoration of p53 tumor suppressor sensitizes *p53*-deficient cancers to mTOR inhibition. Sci. Transl. Med..

[24] Kaczmarek J.C., Patel A.K., Rhym L.H., Palmiero U.C., Bhat B., Heartlein M.W., DeRosa F., Anderson D.G. (2021). Systemic Delivery of mRNA and DNA to the Lung using Polymer-Lipid Nanoparticles. Biomaterials.

[25] Ayad C., Libeau P., Lacroix-Gimon C., Ladavière C., Verrier B. (2021). LipoParticles: Lipid-Coated PLA Nanoparticles Enhanced In Vitro mRNA Transfection Compared to Liposomes. Pharmaceutics.

[26] Mohanty A., Uthaman S., Park I.-K. (2020). Utilization of Polymer-Lipid Hybrid Nanoparticles for Targeted Anti-Cancer Therapy. Molecules.

[27] Mandal B., Bhattacharjee H., Mittal N., Sah H., Balabathula P., Thoma L.A., Wood G.C. (2013). Core–shell-type lipid–polymer hybrid nanoparticles as a drug delivery platform. Nanomed. Nanotechnol. Biol. Med..

[28] Mukherjee A., Waters A.K., Kalyan P., Achrol A.S., Kesari S., Yenugonda V.M. (2019). Lipid–polymer hybrid nanoparticles as a next-generation drug delivery platform: State of the art, emerging technologies, and perspectives. Int. J. Nanomed..

[29] Ahn J., Ko J., Lee S., Yu J., Kim Y., Jeon N.L. (2018). Microfluidics in nanoparticle drug delivery; From synthesis to pre-clinical screening. Adv. Drug Deliv. Rev..

[30] Shepherd S.J., Issadore D., Mitchell M.J. (2021). Microfluidic formulation of nanoparticles for biomedical applications. Biomaterials.

[31] Carugo D., Bottaro E., Owen J., Stride E., Nastruzzi C. (2016). Liposome production by microfluidics: Potential and limiting factors. Sci. Rep..

[32] Niculescu A.-G., Chircov C., Bîrcă A., Grumezescu A. (2021). Nanomaterials Synthesis through Microfluidic Methods: An Updated Overview. Nanomaterials.

[33] Webb C., Forbes N., Roces C.B., Anderluzzi G., Lou G., Abraham S., Ingalls L., Marshall K., Leaver T.J., Watts J.A. (2020). Using microfluidics for scalable manufacturing of nanomedicines from bench to GMP: A case study using protein-loaded liposomes. Int. J. Pharm..

[34] Sun J., Zhang L., Wang J., Feng Q., Liu D., Yin Q., Xu D., Wei Y., Ding B., Shi X. (2014). Tunable Rigidity of (Polymeric Core)-(Lipid Shell) Nanoparticles for Regulated Cellular Uptake. Adv. Mater..

[35] Shen Z., Ye H., Yi X., Li Y. (2018). Membrane Wrapping Efficiency of Elastic Nanoparticles during Endocytosis: Size and Shape Matter. ACS Nano.

[36] Zhong Z., Ji Q., Zhang J.A. (2010). Analysis of cationic liposomes by reversed-phase HPLC with evaporative light-scattering detection. J. Pharm. Biomed. Anal..

[37] Tietje C., Brouder A. (2010). International Conference on Harmonisation of Technical Requirements for Registration of Pharmaceuticals for Human Use. Handbook of Transnational Economic Governance Regimes.

[38] Khadke S., Roces C.B., Cameron A., Devitt A., Perrie Y. (2019). Formulation and manufacturing of lymphatic targeting liposomes using microfluidics. J. Control. Release.

[39] Webb C., Khadke S., Schmidt S.T., Roces C.B., Forbes N., Berrie G., Perrie Y. (2019). The Impact of Solvent Selection: Strategies to Guide the Manufacturing of Liposomes Using Microfluidics. Pharmaceutics.

[40] Karnik R., Gu F., Basto P., Cannizzaro C., Dean L., Kyei-Manu W., Langer R., Farokhzad O.C. (2008). Microfluidic Platform for Controlled Synthesis of Polymeric Nanoparticles. Nano Lett..

[41] Valencia P.M., Pridgen E.M., Rhee M., Langer R., Farokhzad O.C., Karnik R. (2013). Microfluidic Platform for Combinatorial Synthesis and Optimization of Targeted Nanoparticles for Cancer Therapy. ACS Nano.

[42] Hasani-Sadrabadi M.M., Taranejoo S., Dashtimoghadam E., Bahlakeh G., Majedi F.S., VanDersarl J.J., Janmaleki M., Sharifi F., Bertsch A., Hourigan K. (2016). Microfluidic Manipulation of Core/Shell Nanoparticles for Oral Delivery of Chemotherapeutics: A New Treatment Approach for Colorectal Cancer. Adv. Mater..

[43] Lallana E., Donno R., Magrì D., Barker K., Nazir Z., Treacher K., Lawrence M.J., Ashford M., Tirelli N. (2018). Microfluidic-assisted nanoprecipitation of (PEGylated) poly (d,l-lactic acid-co-caprolactone): Effect of macromolecular and microfluidic parameters on particle size and paclitaxel encapsulation. Int. J. Pharm..

[44] Zoqlam R., Morris C.J., Akbar M., Alkilany A.M., Hamdallah S.I., Belton P., Qi S. (2021). Evaluation of the Benefits of Microfluidic-Assisted Preparation of Polymeric Nanoparticles for DNA Delivery. Mater. Sci. Eng. C.

[45] Kim Y., Chung B.L., Ma M., Mulder W.J.M., Fayad Z.A., Farokhzad O.C., Langer R. (2012). Mass Production and Size Control of Lipid–Polymer Hybrid Nanoparticles through Controlled Microvortices. Nano Lett..

[46] Wei W., Sun J., Guo X.-Y., Chen X., Wang R., Qiu C., Zhang H.-T., Pang W.-H., Wang J.-C., Zhang Q. (2020). Microfluidic-Based Holonomic Constraints of siRNA in the Kernel of Lipid/Polymer Hybrid Nanoassemblies for Improving Stable and Safe In Vivo Delivery. ACS Appl. Mater. Interfaces.

[47] Thevenot J., Troutier A.-L., Putaux J.-L., Delair T., Ladavière C. (2008). Effect of the Polymer Nature on the Structural Organization of Lipid/Polymer Particle Assemblies. J. Phys. Chem. B.

[48] Zhang L., Feng Q., Wang J., Zhang S., Ding B., Wei Y., Dong M., Ryu J.-Y., Yoon T.-Y., Shi X. (2015). Microfluidic Synthesis of Hybrid Nanoparticles with Controlled Lipid Layers: Understanding Flexibility-Regulated Cell–Nanoparticle Interaction. ACS Nano.

[49] Teulon J.-M., Godon C., Chantalat L., Moriscot C., Cambedouzou J., Odorico M., Ravaux J., Podor R., Gerdil A., Habert A. (2018). On the Operational Aspects of Measuring Nanoparticle Sizes. Nanomaterials.

[50] Mohammadi S.S., Vaezi Z., Naderi-Manesh H. (2021). Improvement of anti-biofilm activities via co-delivery of curcumin and gentamicin in lipid-polymer hybrid nanoparticle. J. Biomater. Sci. Polym. Ed..

[51] Coolen A.-L., Lacroix C., Mercier-Gouy P., Delaune E., Monge C., Exposito J.-Y., Verrier B. (2018). Poly(lactic acid) nanoparticles and cell-penetrating peptide potentiate mRNA-based vaccine expression in dendritic cells triggering their activation. Biomaterials.

[52] Bose R.J.C., Arai Y., Ahn J.C., Park H., Lee S.-H. (2015). Influence of cationic lipid concentration on properties of lipid–polymer hybrid nanospheres for gene delivery. Int. J. Nanomed..

